# Robotic assistance for percutaneous needle insertion in the kidney: preclinical proof on a swine animal model

**DOI:** 10.1186/s41747-022-00265-1

**Published:** 2022-03-08

**Authors:** Thierry de Baere, Charles Roux, Guillaume Noel, Alexandre Delpla, Frederic Deschamps, Eloi Varin, Lambros Tselikas

**Affiliations:** 1 Department of Interventional Radiology, Gustave Roussy, Villejuif, France; 2 Université Paris-Saclay, Villejuif, France; 3 Department of Pre-Clinical, Biomedical and Analytical Investigations, Biovivo/Claude Bourgelat Institut, VetAgro Sup, Marcy l’Etoile, France

**Keywords:** Ablation techniques, Biopsy (needle), Kidney, Robot-enhanced procedures, Tomography (x-ray computed)

## Abstract

**Background:**

We evaluated the accuracy, safety, and feasibility of a computed tomography (CT)-guided robotic assistance system for percutaneous needle placement in the kidney.

**Methods:**

Fiducials surgically implanted into the kidneys of two pigs were used as targets for subsequent robotically-assisted needle insertion. Robotically-assisted needle insertions and CT acquisitions were coordinated using respiratory monitoring. An initial scan volume data set was used for needle insertion planning defining skin entry and target point. Then, needle insertion was performed according to robot positioning. The accuracy of needle placement was evaluated upon the distance between the needle tip and the predefined target on a post needle insertion scan. A delayed contrast-enhanced CT scan was acquired to assess safety.

**Results:**

Eight needle trajectories were performed with a median procedural time measured from turning on the robotic system to post needle insertion CT scan of 21 min (interquartile range 15.5−26.5 min). Blind review of needle placement accuracy was 2.3 ± 1.2 mm (mean ± standard deviation) in lateral deviation, 0.7 ± 1.7 mm in depth deviation, and 2.8 ± 1.3 mm in three-dimensional Euclidian deviation. All needles were inserted on the first attempt, which determined 100% feasibility, without needle readjustment. The angulation and length of the trajectory did not impact on the needle placement accuracy. Two minor procedure-related complications were encountered: 2 subcapsular haematomas (13 × 6 mm and 35 × 6 mm) in the same animal.

**Conclusions:**

Robotically-assisted needle insertion was shown feasible, safe and accurate in a swine kidney model. Further larger studies are needed.

**Supplementary Information:**

The online version contains supplementary material available at 10.1186/s41747-022-00265-1.

## Key points

- Robotic needle navigation for kidney puncture proved accuracy in an animal model whatever is the angulation and the depth of the puncture.
- Robotic needle navigation for kidney puncture proved to be safe in an animal model.
- Robotic needle navigation can augment the interventional radiologist in percutaneous needle puncture of kidney.

## Background

Thermal ablations of renal cancer have demonstrated impressive outcomes in the treatment of small renal tumours, and are included as a potential treatment in several guidelines by urological and radiological societies [1, 2]. During these treatments, various imaging techniques can be used for image-guided percutaneous insertions of needles and probes into tumours deeply located in the body either for biopsy or thermal ablation as part of routine cancer diagnostic and treatment [3]. Ultrasound has the advantage of real-time monitoring during needle insertion and treatment delivery but is highly impacted by the patient’s morphology. Computed tomography (CT) is extensively used in renal ablation for needle guidance, and needle robotic guidance under CT guidance has recently been developed with the main goal of increasing accuracy, reproducibility, possibility of any angulation, and reducing duration of procedures, number of needle adjustments, learning curve, operators and patient’s exposure to radiations [4–8]. The EPIONE® robotic device (Quantum Surgical, Montpellier, France) is a robotised device that assists the physician during CT-guided percutaneous needle insertion and has recently been CE marked after an early clinical evaluation for liver tumour ablation. We herein report animal evaluation for needle insertion in the kidney.

## Methods

This study was conducted on a total of two swines, in compliance with animal health regulations, Biovivo’s Standard Operating Procedures and the principles of Good Laboratory Practice. The protocol was submitted to the VetAgro Sup Ethics Committee (number 1934).

### Study endpoints

The primary study endpoints were accuracy and safety of needle insertion. Needle insertion accuracy was defined as the distance between the needle tip and the centre of the targeted fiducial. Needle inserted within 5 mm to the targeted fiducial defined the procedure as accurate. The safety evaluation was obtained from procedure-related complications assessed by an interventional radiologist who reviewed the post insertion CT and by the clinical evaluation of a veterinarian. Adverse events were defined according to Common Terminology Criteria for Adverse Event 5.0 [9].

The secondary endpoints were feasibility and adjustment of needle placement. Feasibility was defined as the percentage of successful needle insertions, exempt of problems encountered with the medical device tested during the intervention. A successful placement then allows the procedure to be carried on without reiteration of planning and insertion, after guide release and imaging control. Needle placement adjustment was defined as the number of needle repositioning needed to reach the target.

### Robotic device

**Fig. 1:**
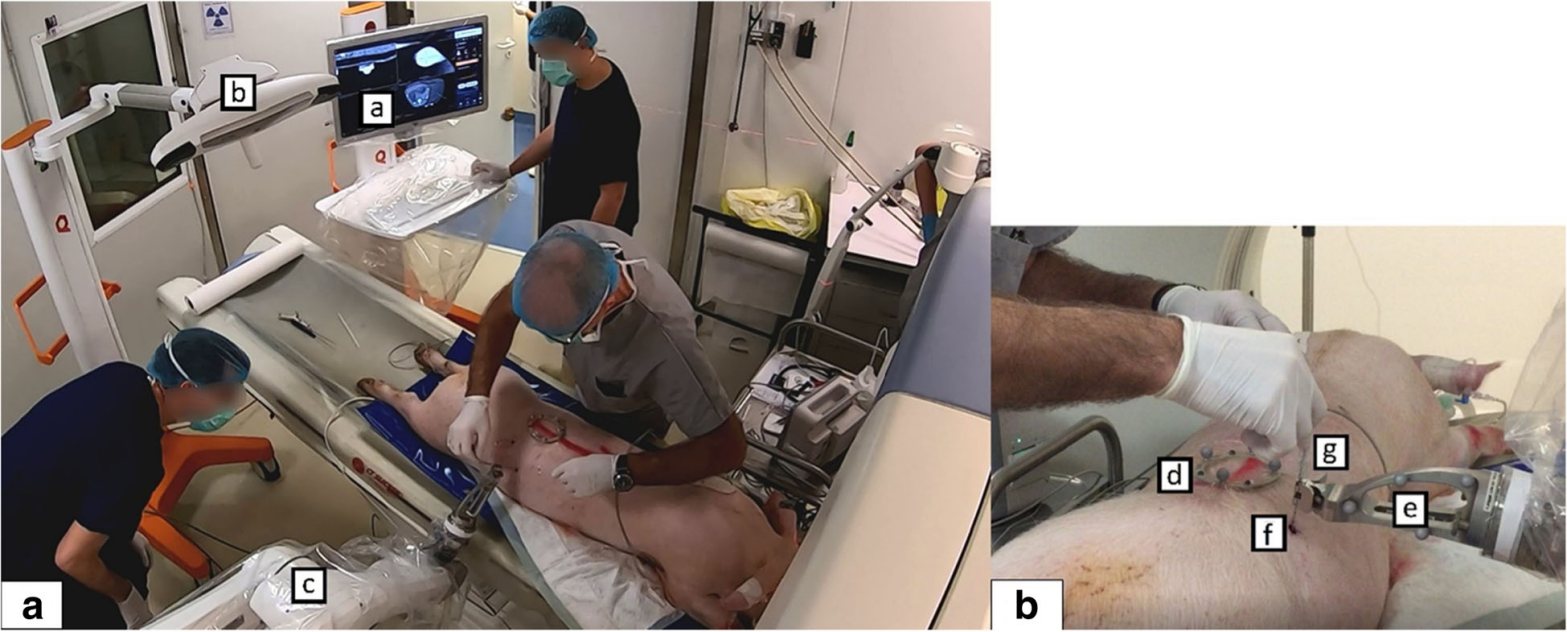
Skyview of the whole new robotic system (**a**) and the operator’s hands during the procedure (**b**). The device is composed of the mobiles display cart (**a**), navigation cart (**b**) and robot cart (**c**). The patient reference (**d**) is attached to the skin of the animal, the robotic arm bears a needle guide (**e**) in which the needle is inserted by the radiologist. The needle guide is automatically positioned at the entry skin point (**f**) for needle (**g**) insertion. The needle is manually inserted inside of the needle guide and pushed until the end position. The needle is then released from the needle guide and the robot arm is manually moved away from the puncture site

The EPIONE® robotic device (Quantum Surgical, Montpellier, France) consists of a mobile cart that carries a robotic arm bearing a needle guide, a mobile display cart, a patient reference (Quantum Surgical, Montpellier, France) attached to the animal skin, that monitors the patient’s motions including breathing, with the help of a mobile navigation camera (NDI Polaris Vega, Ontario, Canada) (Fig. 1a).

### Preparing the animals

Two animals weighing approximately 50 kg were placed under general anaesthesia and received pre-medication with morphine at 0.1 mg/kg (subcutaneous route). Anaesthesia was induced with xylazine at 1.5 mg/kg (intramuscular route) and tiletamine + zolazepam at 3.75 mg/kg (intramuscular route). The animals were then intubated and placed under general anaesthesia using Isoflurane-O_2_ and injection of curare cisatracurium (0.1 to 0.4 mg/kg by intravenous route) renewed as many times as necessary, in order to constrain the breathing movements of the animals. Each animal was surgically implanted with 8 radiopaque fiducials (4 per kidney) measuring 1 mm × 5 mm (GTP 1820≠2, Geotek Medical, Ankara, Turkey) 1 or 2 days before the experiment. Fiducials were surgically inserted to make sure that the robot could target all fiducials wherever they were within the kidney, and not just those that were percutaneously implantable. At the end of the surgery, the animals were awakened.

### CT protocol

All CT scans were performed using a 16-slice Brightspeed CT scanner (General Electric Healthcare, Chicago, IL). Animals were in ventral position and abdomino-pelvic acquisitions were performed with a 35-cm field of view. The scanning parameters were 120 kVp, 150 mA, 0.8 s gantry rotation time, and 0.938 pitch. Scans were reconstructed with a 0.625-mm slice thickness using the ‘SOFT' convolution kernel also provided by General Electric, a standard soft tissue convolution kernel.

### Percutaneous robotically guided needle insertions

#### Needle trajectory planning

A pre-puncture CT volume was acquired under apnoea and loaded into the robotic device software. Then, the needle trajectory was defined in order to reach the centre of a fiducial and to avoid bone and critical structures in the abdomen (Video 1 in Supplementary Material). Among the four fiducials implanted in each kidney, two fiducials were selected in each kidney and thus the procedure was repeated twice per kidney.

#### Robotically-assisted needle insertion

All procedures were performed by one experienced interventional radiologist (more than 10 years of experience in image-guided procedures). The robot arm was sent to its predefined ‘HOME’ position. The needle guide was attached to the robot arm. Then, the software automatically located the ‘patient’ reference markers in the images. The position of each marker was verified and manually adjusted whenever necessary. A 17-gauge disposable coaxial biopsy needle (Bard Medical, Covington, USA) with the appropriate needle length (from 10.6 to 14.7 cm), *i.e.*, a needle with a length slightly greater than the trajectory length, was selected into the planning software. The robot arm was manually brought close to the area of interest in order to place both the ’patient’ reference and the needle guide within the optical camera’s field of view. Then, the robot arm automatically positioned the needle guide in line with the planned trajectory. After the skin incision was made at the needle entry point, the needle was manually inserted inside of the needle guide and pushed until the end position (Supplementary material: Video 2 and Fig. 1b). The needle was then released from the needle guide and the robot arm was manually moved away from the puncture site.

#### Needle placement verification

A CT scan image was acquired under apnoea to assess needle placement accuracy. The procedure was deemed complete when the operator judged the needle tip as being ≤ 5 mm of the target. Otherwise, an additional attempt was allowed with the same planning. A third attempt was not permitted.

### Safety assessment

Five minutes after needle removal, a contrast-enhanced CT scan of the abdomino-pelvic area was performed 60 s after the injection of 30 mL of ioxehol (Omnipaque®350 mg/mL, General Electrics Healthcare, Chicago, IL) at a rate of 2.2 mL/s. Any procedure-related complication that can be depicted on this CT scan was reported.

### Apnoea repeatability test

To verify that the kidney was repositioned at the same location under repeated apnoea, a repeatability test was performed on all the fiducials inserted into the two swines, using the respiratory monitoring module. Apnoea was induced during all CT scan acquisitions and robotically-assisted needle insertions by turning off the ventilation at the end of expiration.

Two successive unenhanced CT scans were acquired during apnoea to assess their repeatability. In-between, animal breathing was resumed. The location of each fiducial was determined on the two CT scans, using Digital imaging and communication in medicine (DICOM) coordinates with a standard DICOM image viewer software (Myrian v2.4, Intrasense, Montpellier, France; https://intrasense.fr/fr/). The coordinates of each fiducial relative to the patient reference were determined and the three-dimensional (3D) Euclidean deviation between the two acquisitions was computed to determine the displacement of the fiducial under repeated apnoea. Apnoeas were considered as repeatable if the displacements of the 8 radiopaque fiducials were below 2 mm in-between the two subsequent CT scans. The displacement of the fiducials under subsequent apnoea was calculated in relation to the ‘patient’ reference in order to strictly reproduce the operating principle of the robotic system.

### Statistical analysis

For this preliminary study, no specific sample size calculations were performed. The description of the relevant study variables was performed using, mean, standard deviation, median, min and max for quantitative variables and using frequency and percentage for qualitative variables.

The needle placement accuracy was determined by calculating the 3D Euclidean deviation between the needle tip position and the centre of the fiducial (targeted point). The Myrian® Image viewer (Myrian v2.4, Intrasense, Montpellier, France; https://intrasense.fr/fr) was used to obtain the coordinates of the points in the DICOM images and to evaluate the 3D Euclidian deviation, the lateral deviation and the depth deviation. These deviations were described as quantitative variables.

All measurements were performed blindly by an experienced interventional radiologist. 3D deviation, lateral deviation and depth deviation least square mean estimates (and 95% upper limit) were provided using a random effect model for the animal; *p* value testing for the inferiority of mean to 5 mm was calculated.

Exploratory analyses were performed to identify potential factors influencing the needle placement accuracy (angulations, skin entry to target distance).

All attempts were prospectively included in the statistical analysis. There was no missing data imputation. All statistical analyses were produced using SAS® 9.4 software (Cary, NC, USA).

## Results

### Robotically-assisted needle insertion

A total of 8 needle insertions with 8 different trajectories were planned and executed. Considering the 8 trajectories, the median [min; max] length of the needle path from entry skin to target was 52.5 [31; 73] mm. The median [min; max] orbital angulation was -6.0° [-59.8°; 28.5°] and the median [min; max] craniocaudal angulation was 16.8° [-0.8°; 52.7°]. Seven (87.5%) of trajectories were out of plane (≥ 5° in the *z*-axis). The median [min; max] procedure time from the start of the trajectory (turning on the EPIONE® device) to visual verification on the CT scan acquired for needle placement evaluation was 21 [13; 35] min (Table 1).

**Table 1:** Characteristics of needle insertions

Parameter	***n***	Mean (SD)	Median (Q1; Q3)	Min; Max
Distance from skin entry point to target (mm)	8	50.9 (13.8)	52.5 (39.8; 59.5)	31.0; 73.0
Orbital angulations (°)	8	-11.1 (26.8)	-6.0 (-26.8; 3.9)	-59.8; 28.5
Cranio-caudal angulations (°)	8	21.3 (19.1)	16.8 (6.9; 35.6)	-0.8; 52.7
Time from the beginning of the procedure (turning on the device) to needle placement (min)	8	21.8 (7.5)	21.0 (15.5; 26.5)	13.0; 35.0

*Max* Maximum, *Min* Minimum, *Q1* Inferior quartile, *Q3* Superior quartile, *SD* Standard deviation

**Fig. 2:**
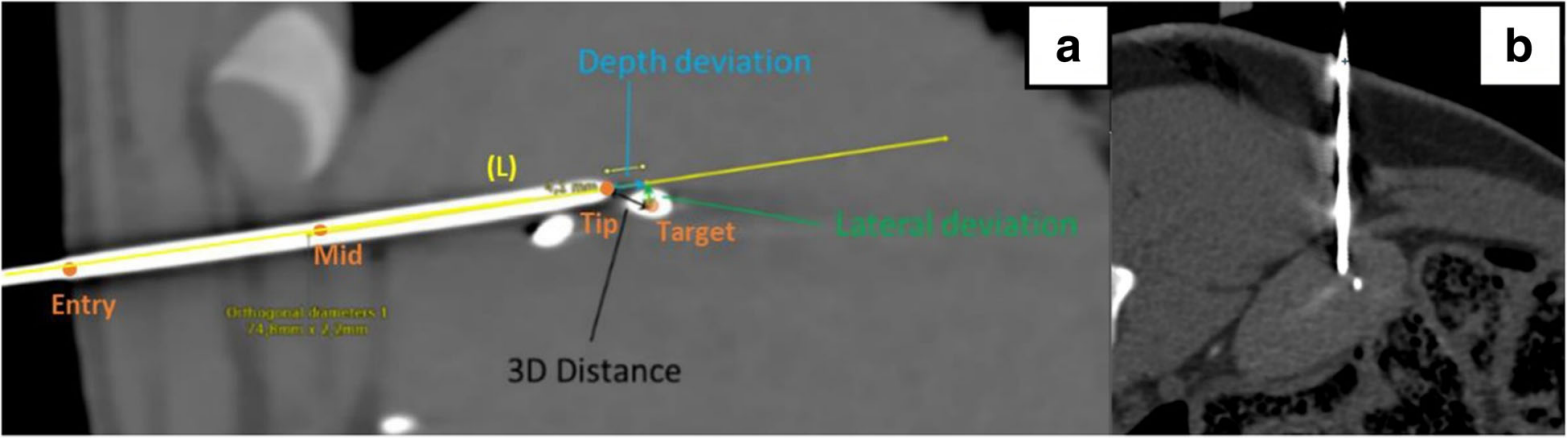
Technique of evaluation of accuracy based on a CT obtained after needle insertion. Reconstruction along the needle axis in the plane of the centre of the targeted fiducial is used for measurement of depth deviation, lateral deviation and three-dimensional distance (**a**). Typical multiplanar reconstruction along needle axis in the fiducial plane for evaluation of needle placement accuracy (**b**)

All 8 fiducials were accurately targeted on the first attempt according to the visual evaluation of the operator. Blind review measurements revealed a mean (standard deviation [SD]) 3D deviation of 2.8 (1.3) mm, the means (SD) lateral deviation and depth deviation were 2.3 (1.2) mm and 0.7 (1.7) mm, respectively. Details of needle placement accuracy measurements and results are provided in Fig. 2 and Table 2.

**Table 2:** Needle placement accuracy: summary of 3D, lateral, and depth deviations

Parameter	***n***	Mean (SD)	Median (Q1; Q3)	Min; Max	LS Mean [95% upper limit]	***p*** value*
3D deviation (mm)	8	2.80 (1.31)	2.68 (1.75; 3.88)	1.18; 4.64	2.73 [0; 6.79]	0.0877
Lateral deviation (mm)	8	2.27 (1.18)	2.16 (1.40; 3.02)	0.74; 4.28	2.27 [0; 3.06]	0.0002
Depth deviation (mm)	8	0.70 (1.69)	1.02 (-0.42; 1.83)	-2.25; 2.99	0.70 [0; 1.83]	<.0001

*3D* Three-dimensional, *Max* Maximum, *Min* Minimum, *Q1* Inferior quartile, *Q3* Superior quartile, *SD* Standard deviation

*p value for unilateral testing distance H0 >5 mm *versus* H1 ≤ 5 mm. Least square (LS) mean and 95% upper limit taking into account intra-animal and fiducial nested in animal variability structure

Neither orbital angulation, craniocaudal angulation, nor trajectory length had an impact on the accuracy of needle placement.

### Safety assessment

Among the 8 needle insertions, two subcapsular haematomas (measuring 13 × 6 mm and 35 × 6 mm) occurred in the same animal. Haematomas remained subcapsular without retroperitoneal effusion and without any haemodynamic consequences. No other side effects were found including urine leakage. Consequently, these localised haematomas were considered as grade 1 adverse events according to the Common Terminology Criteria for Adverse Events, CTCAE, version 5.0 [9].

### Apnoea repeatability test

Per-fiducial displacement comparison showed that all 16 fiducials depicted on CT moved less than 2 mm between two consecutive apnoeas. The 3D deviation of the fiducials between CT-scans acquired during two consecutive apnoeas was significantly lower than 2 mm (*p* = 0.019) with a least-squares mean of 0.5 mm and a 95% upper limit of 1.1 mm.

## Discussion

Accuracy of needle placement is a key parameter for the success of percutaneous biopsy or thermal ablation. In our experiment, the 3D Euclidean deviation from the needle tip to the target ranged from 1.2 to 4.6 mm, with a mean (SD) of 2.8 (1.3) mm highlighting that we met the high accuracy needed to make biopsy and thermal ablation reliable. Very few reports on robotic navigation in the kidney are available for comparison. Our results are comparable with the subpopulation of kidney puncture in Hiraki et al. multiorgan experiment, where the mean (SD) of needle placement accuracy in the kidney was 2.9 (1.0) mm [10] . Moreover, as outlined by Engstrand et al., the needle lateral deviation can be considered as the most important parameter to consider when evaluating the accuracy, because it is complex to compensate and most often requires a new puncture or needle redirection [11], while the depth error can be compensated by pushing or retrieving the needle on the same puncture tract. In the present study, the lateral deviation was considered low, from 0.7 to 4.3 mm, traduced by an impressive 0% needle adjustment or second puncture, particularly when compared to other CT-guided robotic needle placement in the kidney, which reported a median of 2 to 4 needle adjustments [10].

In the present study, angulations of needle trajectories in the *z*-axis ranged from -59.8° to + 28.5°. These angulations neither impacted accuracy nor procedure duration.

Seven out of 8 (87.5%) trajectories were out of plane (≥ 5° in the *z*-axis), thus countering one of the CT limitations in guiding needle insertion when outside the axial plane identified by Wood et al. [12]. Indeed, visualisation of the full length of needles outside the axial plane is difficult on CT imaging as several axial planes must be reviewed to visualise the full length of the needle, or volume with multiplanar reconstruction must be performed thus losing the nearly real-time ability of the CT and increasing irradiation [13]. Slight angulation can be managed with an associated gantry angulation when possible. On the other hand, obtaining angulation with the gantry has the effect of limiting access to the patient and making it impossible to insert long needles [14]. Therefore, the common practice is to limit the needle trajectory to the axial plane or very limited cranio-caudal angulation.

Currently, patient and physician irradiation is unfortunately part of CT-guided needle insertions [4, 15–18]. In this preclinical experience, only two CT acquisitions were required for needle placement: a CT for planning and a CT to confirm needle position. The needle was inserted on breath hold without additional x-rays exposures. Even though animal and operator radiation exposure doses were not measured, operator irradiation was nil, and it is expected to reduce patient irradiation with the use of the robot. A study comparing freehand *versus* robotic insertion has shown a significantly higher total radiation dose of 1075.77 mGy/cm and 636.4 mGy/cm for freehand and robotic needle insertions, respectively; this is understandable, due to the need of more confirmatory scans and needle manipulations for freehand procedures [19].

Regarding safety, we recorded two grade 1 procedure-related haematomas among eight trajectories, which is similar to what was previously reported by others for pig, with one pararenal haematoma out of five punctures [6, 10].

The system we evaluated offers no compensation for target motion or needle bending [15], integrating closed-loop active compensation that is expected to improve targeting accuracy with user-directed cooperative insertion [20]. We believe that fast and simple needle insertion has advantages over repetitive imaging and that straight-line insertion minimises the risk of organ displacement. Tissue deformation and target displacement by the needle itself appears to be minimal as shown by our accuracy measurements. It can be assumed that when a puncture is performed freehand, the needle undergoes some redirections which alone may displace organs and targets [19–22]. A key feature of the device we used is that the respiratory monitoring module was able to deal with the respiratory motion in the context of general anaesthesia with mechanical ventilation as confirmed by our apnoea repeatability test. This tool is especially useful and impacting on needle placement accuracy [23].

This study has limitations. Only 17-gauge biopsy needles with low bending capacity were used while punctures with smaller bevelled calibre needles may be more prone to bending. However, Hiraki et al. demonstrated that there is no significant difference in insertion accuracy according to the type of needle used [10]. Moreover, the tip of these kinds of needles is very sharp, which may not be the case for ablation needles in clinical cases. Another limitation of this pilot study is the lack of comparison between the accuracy of robotic-assisted procedures and freehand insertion. It would be of interest to compare freehand placement to robotically assisted placement and study if operator experience may have a significant impact on accuracy, outcome, and complication rates. At last, a single operator performed all the insertions, although, we previously demonstrated that accuracy did not differ between novice and experienced operators [24], and we do believe that when using robotic, very little is left to operator experience. Finally, one of the limitations of the study is that general anaesthesia was required for apnoea because sedation is not possible on an animal and because the system today is dedicated to thermal ablation. Further evaluations are needed in clinical practice to determine whether patient self-apnoea validated by the apnoea repeatability test embedded in our system can allow for accurate robotic navigation of needles.

Today ultrasound is one of the most popular imaging modalities for needle insertion in the liver and kidney due to its availability and real-time ability. However, some drawbacks exist and notably, patient morphology and deeply located tumours are difficult to see even with the help of contrast-enhanced ultrasound. CT can be used but it is a not real-time imaging modality, have some limitations outside of the axial plane, and even if contrast enhancement allows good conspicuity of the targeted tumour, such enhancement might be fleeting and thus not present during all procedure of needle insertion. MRI is hardly available to guide needle insertion. Renal ablation in a dedicated setting, including both US and CT has been reported, in order to merge the benefit of both techniques [25]. Numerous publications on fusion imaging applied to thermal ablations have been produced in the recent years highlighting the difficulty of a single technique. Fusion imaging has been reported to increase the conspicuity of targeted tumours but has some difficulties obtaining accurate registration [26, 27]. The robotic system allows to use a single CT acquisition with maximum enhancement to plan needle insertion and treatment and allows for excellent spatial reproducibility as demonstrated by our apnoea reproducibility. Of course, we do not provide real-time monitoring, but it is possible, if needed, to obtain post-ablation CT imaging for confirmation of ablation safety margins.

In conclusion, we provided additional data increasing a scarce literature about robotic needle insertion, by reporting a 100% successful and accurate needle placement in the kidney in any needle tract angulation without major complications. These promising results will challenge freehand needle placement by senior interventional radiologists and allow for high accuracy to less experienced operators or centres, when local treatment of small size renal cancer is a challenge in an ageing population. These results suggest that robotic-assisted needle guidance is part of the future of interventional oncology and must be mastered in order to provide safe, accurate, and reproducible diagnostic and therapeutic interventional oncology procedures to cancer patients.

## Supplementary Information

**Additional file 1.** Video 1 : Needle trajectory planning.

**Additional file 2.** Video 2 : Robotically-assisted needle insertion.

## Data Availability

The datasets used and/or analysed during the current study are available from the corresponding author on reasonable request.

## Abbreviations

3D: Three-dimensional
CT: Computed tomography
DICOM: Digital imaging and communication in medicine
SD: Standard deviation
